# Genome-Wide Gene-Based Multi-Trait Analysis

**DOI:** 10.3389/fgene.2020.00437

**Published:** 2020-05-19

**Authors:** Yamin Deng, Tao He, Ruiling Fang, Shaoyu Li, Hongyan Cao, Yuehua Cui

**Affiliations:** ^1^Division of Health Statistics, School of Public Health, Shanxi Medical University, Taiyuan, China; ^2^Department of Mathematics, San Francisco State University, San Francisco, CA, United States; ^3^Department of Mathematics and Statistics, University of North Carolina at Charlotte, Charlotte, NC, United States; ^4^Department of Statistics and Probability, Michigan State University, East Lansing, MI, United States

**Keywords:** gene-based association, kernel function, multi-trait, nonlinear effect, *p*-value combination

## Abstract

Genome-wide association studies focusing on a single phenotype have been broadly conducted to identify genetic variants associated with a complex disease. The commonly applied single variant analysis is limited by failing to consider the complex interactions between variants, which motivated the development of association analyses focusing on genes or gene sets. Moreover, when multiple correlated phenotypes are available, methods based on a multi-trait analysis can improve the association power. However, most currently available multi-trait analyses are single variant-based analyses; thus have limited power when disease variants function as a group in a gene or a gene set. In this work, we propose a genome-wide gene-based multi-trait analysis method by considering genes as testing units. For a given phenotype, we adopt a rapid and powerful kernel-based testing method which can evaluate the joint effect of multiple variants within a gene. The joint effect, either linear or nonlinear, is captured through kernel functions. Given a series of candidate kernel functions, we propose an omnibus test strategy to integrate the test results based on different candidate kernels. A *p*-value combination method is then applied to integrate dependent *p*-values to assess the association between a gene and multiple correlated phenotypes. Simulation studies show a reasonable type I error control and an excellent power of the proposed method compared to its counterparts. We further show the utility of the method by applying it to two data sets: the Human Liver Cohort and the Alzheimer Disease Neuroimaging Initiative data set, and novel genes are identified. Our method has broad applications in other fields in which the interest is to evaluate the joint effect (linear or nonlinear) of a set of variants.

## Introduction

Methods on genome-wide association studies (GWAS) are mostly focused on single variant (e.g., single nucleotide polymorphism, SNP) analysis with a single phenotype, the so-called single-variant single-trait analysis. Increasing evidence shows that pleiotropy, the effect of one gene on multiple phenotypes (often correlated), plays a pivotal role in many complex traits (Stearns, 2010; Schifano et al., 2013). For example, cognitive ability is often assessed in many domains such as memory, intelligence, language, and visual–spatial function (Yang and Wang, 2012). Instead of analyzing one trait at a time, we can take the correlated structure of multiple phenotypes into account and analyze them in a multi-trait analysis. As a complementary approach, such type of analysis can not only gain association power by aggregating multiple weak signals (He et al., 2013; Schifano et al., 2013; Wang, 2014) but also lead to better understanding of disease etiology by detecting genetic variants with pleiotropic effects (Amos and Laing, 1993; Jiang and Zeng, 1995; Schifano et al., 2013).

For a multi-trait analysis, one commonly applied method is the one-way multivariate analysis of variance (MANOVA) (Bilodeau, 2013). Unfortunately, most multi-trait data do not satisfy the multivariate normal assumption for MANOVA, hence greatly limiting its applicability. Other methods are developed based on the idea of dimension reduction. For example, a multivariate response can be summarized into a univariate score using principal component (PC) analysis, based on which traditional univariate association methods can be applied (e.g., Zhang et al., 2012). As the first PC contains the most information about multiple phenotypes, this can change the test between a SNP and multiple phenotypes into a univariate test of association between a SNP and the first PC. The downside for this analysis is the lack of interpretability. Methods focusing on summary statistics have gained much popularity recently since the individual-level data are typically unavailable (e.g., Kim et al., 2015; Turley et al., 2018). However, such methods are largely undermined if the published GWAS summary statistics have limited accuracy. In addition, the marginal SNP effect is usually quite small in many complex diseases, and many identified SNPs have limited biological interpretation, for example, SNPs identified in non-coding regions.

These limitations motivated the development of gene- or pathway-based association analysis aimed at improving the statistical power and gaining novel insight into disease etiology (Wang et al., 2007; Cui et al., 2008; Liu et al., 2010). Firstly, the gene- or pathway-based analysis can largely alleviate the multiple testing burden by more than 10 or 100 folds. Secondly, due to allelic heterogeneity, most diseases are associated with a set of SNPs at different loci, making it hard to replicate the results based on a single-SNP analysis (Neale and Sham, 2004). In this case, a gene- or pathway-based analysis may provide additional insight to reveal the functional mechanism of complex diseases (Wang et al., 2010). Unlike the heterogeneity of a single locus, the biological function of genes is more consistent across populations, which enhances the likelihood of replication (Neale and Sham, 2004; Wang et al., 2010).

Most reports in the literature on multi-trait analysis are focused on a single-variant analysis, which shares the same limitation as described for the single-trait GWAS. Although methods for gene-based analysis focusing on a single trait have been developed, multi-trait analysis focusing on genes or gene sets is largely under-developed. There is a pressing need to develop a gene-based method for a multi-trait analysis.

In a gene-based single-trait analysis, the kernel-based testing (KBT) method is gaining much popularity recently due to its power and flexibility in capturing potential nonlinear effects (Kwee et al., 2008; Mukhopadhyay et al., 2010; Wu et al., 2010; Li and Cui, 2012; Lin et al., 2013; Marceau et al., 2015; Wei and Lu, 2017). The power of the KBT methods depends on the choice of kernel functions which measure the similarity between individuals across multiple genetic variants in a gene. When the underlying true disease function is unknown, this limits the applicability of the KBT methods since the choice of the kernel function needs to be determined. Given a series of candidate kernel functions under the KBT framework, a common method is to choose the kernel function leading to the smallest *p*-value. This idea, however, could inflate the type 1 error rate due to the greedy process of kernel selection. We recently proposed a nonparametric KBT testing procedure which relaxes the distributional assumption required in most KBT methods (He et al., 2019). The asymptotic distribution of the test statistics approximately follows a normal distribution when the number of SNP variants in a gene set, *p*, is large. In fact, the normal approximation works well under a large *p* setting. Given a series of candidate kernel functions, we provided an analytical procedure to evaluate the *p*-value of the maximum statistics.

Based on empirical studies, the approximation method could be underperformed when *p* is relatively small. In this work, we borrowed the same idea but relaxed the large *p* assumption required for the normal approximation and proposed an omnibus testing procedure when multiple candidate kernels are available. Obtaining a *p*-value needs almost negligible computation and can be extremely fast. When extending the method to a multi-trait analysis, we adopted a Fisher *p*-value combination (FPC) method with correlated dependent variables, as proposed by Yang et al. (2016). The FPC provides an alternative approach for multi-trait analysis by integrating the single-trait analysis results. The proposed Omnibus Multi-trait Gene-based Association (OMGA) analysis can capture linear or nonlinear effects without kernel selection and is computationally efficient.

We conduct extensive simulation studies to evaluate the type I error control and power and further compare it with its counterparts. We demonstrate the performance of our proposed method through two real data applications of the Human Liver Cohort (HLC) study and the Alzheimer Disease Neuroimaging Initiative (ADNI) study. The results tell which genes are specific to a single phenotype or contributed to a common genetic construction of multiple phenotypes. Our OMGA method enriches the literature of genome-wide gene-based multi-trait association analysis and has broad applications in other fields where the interest is to evaluate the joint effect (linear or nonlinear) of a set of variants.

## Statistical Methods

### Gene-Based Association Test Based on a Single Trait

#### The Model

To model the association between a gene and a quantitative trait, we consider the following semiparametric model (He et al., 2019),

(1) $$Y_{i}=\mu +\alpha^{T}\,W_{i}+h\,\left(x_{i}\right)+\varepsilon_{i},i=1,2,\ldots .\ldots ,n,$$

where *Y*_*i*_ is the response variable for the *i-*th individual, *n* is the sample size, α is the effect corresponding to *W*_*i*_ = (*W*_*i*1_,*W*_*i*2_,……*W*_*iH*_)^*T*^, a vector of *H*-dimensional covariates containing variables such as age and gender, *x*_*i*_ = (*x*_*i*1_,……*x*_*ip*_)^*T*^ is a vector of a *p*-dimensional SNP set in a given gene where *p* can be large, *h*(⋅) is an unknown function that captures the joint effect of multiple variants in a given SNP set, and ε*_*i*_* is the random error with mean 0 and variance σ^2^. Here, we relax the error distribution assumption for the error term which does not have to follow a normal distribution.

Following model (1), assessing the effect of multiple variants in a given SNP set (e.g., a gene) is equivalent to test the hypotheses *H*_*0*_: *h*(⋅) = 0, while adjusting for the effects of covariates. Wu et al. (2011) proposed a kernel-based test by considering the joint effect of multiple SNPs in a given set and showed great power compared to a multiple-regression approach. In Wu et al. (2011), the function *h*(⋅) is modeled as a random effect and *h*(⋅)∼*N*(0,τ^2^*K*) where τ^2^ is the variance and *K* is a kernel matrix which measures the similarity between individuals across multiple SNP variants. However, the normality assumption on *h*(⋅) limits its power when this assumption is violated. To relax this assumption, He et al. (2019) proposed a U-statistic defined as:

$$T_{n}=\frac{1}{n\,\left(n-1\right)}\,\sum_{i\neq j}K\,\left(X_{i},X_{j}\right)\,\left(Y_{i}-{\hat{Y}}_{i}\right)\,\left(Y_{j}-{\hat{Y}}_{j}\right)/{\hat{\sigma }}^{2},$$

where $\hat{Y}$ and ${\hat{\sigma }}^{2}$ are sample estimates under the null model *Y*_*i*_ = μ + α^*T*^*W*_*i*_ + *ε*_*i*_; $K\,\left(X_{i},X_{j}\right)=\frac{K_{\theta }\,\left(X_{i},X_{j}\right)}{\sqrt{E\,\left\{K_{\theta }\,\left(X_{i},X_{i}\right)\right\}\,E\,\left\{K_{\theta }\,\left(X_{j},X_{j}\right)\right\}}}$ is the normalized kernel for kernel *K*_θ_(*X*_*i*_,*X*_*j*_). In practice, the choice of kernel function for *K*_θ_(*X*_*i*_,*X*_*j*_) depends on the underlying relationship between SNPs and the disease response. For example, a linear kernel is applied if the relationship between multiple SNP variants and the disease response is linear, and a Gaussian or polynomial kernel can be applied if a nonlinear relationship between multiple SNPs and the disease response is assumed. Several widely used kernel functions include the linear kernel $K_{\theta }\,\left(X_{i},X_{j}\right)=X_{i}^{T}\,X_{j}/\theta$, IBS kernel for discrete SNP genotype data $K_{\theta }\,\left(X_{i},X_{j}\right)=\sum_{k=1}^{p}\left(2-\left|X_{i\,k}-X_{j\,k}\right|\right)/2\,p$, and Gaussian kernel *K*_θ_(*X*_*i*_,*X*_*j*_) = *exp*⁡(−||*X*_*i*_−*X*_*j*_||^2^/θ). These kernels will be our candidate kernels in the simulation and real data analysis.

Let ${\tilde{W}}_{n\times \left(L+1\right)}=\left[1_{n},W_{n\times L}\right]$ and $A=\tilde{W}\,{\left({\tilde{W}}^{T}\,\tilde{W}\right)}^{-1}\,{\tilde{W}}^{T}$. Then, we have ${\hat{\sigma }}^{2}=Y^{T}\,\left(I-A\right)\,Y/\left(n-L-1\right)$ and $\hat{Y}=\text{AY}$. Following the Eigen-decomposition, $K\,\left(X_{i},X_{j}\right)=\sum_{m=1}^{\infty }\lambda_{m}\,\phi_{m}\,\left(X_{i}\right)\,\phi_{m}\,\left(X_{j}\right)$ where λ*_*m*_* is the eigenvalues and ϕ_*m*_(⋅) is the orthonormal eigenvectors of the kernel *K*. For any positive integer *k*, let $V_{k}=\sum_{m=1}^{\infty }\lambda_{m}^{k}$. Then, under the null hypothesis of no association, the asymptotic distribution of the test statistic *T*_*n*_ follows a chi-square distribution, i.e.:

$$n\,T_{n}/V_{1}\overset{d}{\to }\sum_{m=1}^{\infty }\lambda_{k,m}\,\left(x_{1,m}^{2}-1\right),$$

where $x_{1,m}^{2}$ are independent chi-square distributions with one degree of freedom. Then, we can apply a Satterthwaite approximation to the mixture of chi-squares by a scaled chi-square distribution $\hat{a}\,\chi_{\hat{g}}^{2}/{\hat{V}}_{1}-1$, where $\hat{g}={\hat{V}}_{1}/\hat{a}$, $\hat{a}={\hat{\sigma }}_{T_{n}}^{2}/2\,{\hat{V}}_{1}$, and ${\hat{V}}_{1}=n^{-1}\,\text{tr}\,\left(\mathrm{HK}\right)$ is a consistent estimator of *V*_1_ with **H** = **I**−*n*^−1^**J** as a projection matrix. Then, an asymptotic *α*-level test rejects the null if

$$\left(n\,T_{n}+{\hat{V}}_{1}\right)/\hat{a}>\chi_{\hat{g},1-\alpha }^{2},$$

where $\chi_{\left(\hat{g},1-\alpha \right)}^{2}$ is the (1 – α)th quantile of a chi-square distribution with $\hat{g}$ degrees of freedom. Following He et al. (2019), $\sigma_{T_{n}}^{2}$ can be estimated by

$$1/n^{2}\left(2-\frac{12}{n^{2}}+\frac{6\,\hat{\Delta }}{n}\right)tr\left(HKHK\right)$$

$$\;-\left(\frac{2}{n}+\frac{\hat{\Delta }}{n}\right)tr^{2}\left(HK\right)+\hat{\Delta }tr\left(B\circ B\right),$$

where ∘ represents the Hadamard product, $\hat{\Delta }=n^{-1}\,\sum_{i=1}^{n}{\left[\frac{{\left(Y\right)}_{i}-{\bar{Y}}_{n}}{\hat{\sigma }}\right]}^{4}-3$, and **B** = **HKH**. Then, the *p*-value of *T*_*n*_ can be obtained.

#### An Omnibus Test With Multiple Candidate Kernels

The method described above works for a given kernel function. There are various kernel functions available to use. For example, if a linear relationship is assumed, then one can apply a linear kernel, while a Gaussian kernel can be applied when potential nonlinear relationship exists. Thus, the power of the proposed test statistic largely depends on the choice of the kernel function. If the optimal kernel function that captures the underlying true relationship cannot be determined, the testing power will suffer. In practice, the true relationship is generally unknown, so does the choice of the kernel function.

To overcome the issue of selecting the optional kernel function, we propose an omnibus test strategy in this work. Given a set of *L* candidate kernels denoted by *K*_1_(⋅,⋅),*K*_2_(⋅,⋅),⋯,*K*_*L*_(⋅,⋅),, we can apply the proposed method and get the corresponding *p*-value denoted by *p*_1_, *p*_2_,…*p*_*L*_. These *L* kernel functions can come from a wide range of choices, such as the linear kernel, the Gaussian kernel, and the polynomial kernel. Then, we can transform the *L p*-values by a Cauchy transformation and combine the transformed *p*-values to form a new statistic (Liu et al., 2019),

$$T_{O}=\frac{1}{L}\,\sum_{j=1}^{L}\tan\left\{\left(0.5-p_{j}\right)\,\pi \right\}.$$

If *p*_*j*_ comes from the null hypothesis, the transformation *tan*⁡{(0.5−*p*_*i*_)π} follows a Cauchy distribution. Then, the *p*-value of *T*_*O*_ can be approximated by

$$p\,\text{-value}\approx 0.5-\left\{\arctan\left(T_{O}\right)\right\}/\pi$$

This Cauchy combination method performs similarly as the minimum *p*-value method. In addition, it works well under different correlation structures. Thus, when the underlying true relationship is unknown, if the choice of the kernel function is rich enough, we can always achieve good power regardless of the underlying disease gene action mode. More importantly, this method is computationally fast and robust to different dependence structures between *p*-values (Liu and Xie, 2019).

### Gene-Based Association Test With Multiple Traits

When multiple correlated traits are available, it is more powerful to analyze them together to find the disease–gene association. One way to do so is to perform a multivariate analysis by treating multiple traits as a multivariate response. Generally speaking, it is much easier to conduct a univariate association test than a multivariate association test. Suppose there are a total of *d* quantitative traits. For a given gene, we can get *d* gene-level *p*-values, denoted by *p*_1_, *p*_2_,…*p*_*d*_. Since these *d* traits are generally correlated and the *p*-values are obtained based on the same gene, these *p*-values are typically correlated. To obtain a gene-based *p*-value for multiple traits, one simple way is to do a *p*-value combination. Unfortunately, the aforementioned Cauchy combination method does not work well in many cases since it functions like a minimum *p*-value approach, and this is not the intention for multi-trait analysis.

When the *d* p-values are independent, the Fisher combination method defined as $T=-2\,\sum_{j=1}^{d}\log\left(p_{j}\right)$ follows a chi-square distribution with 2*d* degrees of freedom (Littell and Folks, 1971). For correlated traits, this method cannot be directly applied to find the association between one gene and multiple traits. In fact, the statistic *T* is a sum of correlated chi-square statistics which can be approximated by a scaled chi-square distribution $\delta \,x_{\tau }^{2}$ or a gamma distribution with a scale parameter of 2δ and a shape parameter of τ/2 under the null hypothesis (Yang et al., 2016). Let *E*(*T*) = μ and Var(*T*) = σ^2^. Then, δ and τ can be computed as δ = σ^2^/2μ and τ = 2μ^2^/σ^2^. Here we adopt the method proposed by Yang et al. (2016) to combine the *d*-dependent *p*-values. The variance σ^2^ can be calculated as

$$\sigma^{2}=V\,a\,r\,\left[T\right]=V\,a\,r\,\left\{-2\,\sum_{j=1}^{d}\log\left(p_{j}\right)\right\}=\sum_{j=1}^{d}V\,a\,r\,\left\{-2\,\log\left(p_{j}\right)\right\}+\sum_{j\neq k}c\,o\,v\,\left(-2\,\log\left(p_{j}\right),-2\,\log\left(p_{k}\right)\right)=4\,d+\sum_{j\neq k}c\,o\,v\,\left(-2\,\log\left(p_{j}\right),-2\,\log\left(p_{k}\right)\right)$$

Let δ_*jk*_ = *cov*{−2*log*⁡(*p*_*j*_),−2*log*⁡(*p*_*k*_)}. Yang et al. (2016) proposed a method to estimate δ_*jk*_ based on which we can estimate σ^2^ [please refer to Yang et al. (2016) for the technical details of estimating σ^2^ and μ]. An R package implementing the method can be found at https://github.com/jjyang2019/FisherCombinationStat. Then, based on the estimators of μ and σ^2^ for the gamma distribution parameters, the overall testing *p*-value of *T* can be calculated as

$$p\,\text{-value}=1-\Gamma \,\left(\mu^{2}/\sigma^{2},\sigma^{2}/\mu \right).$$

The number of the gene-level test is much smaller than the number of the SNP-level test. After obtaining the gene-level *p*-values, multiple testing adjustment such as FDR can be applied to claim the significance of a gene.

## Simulation Studies

### Simulation Design

To evaluate the statistical power and the type 1 error rate of the proposed method, we conducted extensive simulation studies to compare the proposed method (OMGA) with some existing methods. Specifically, we compared with the method of multivariate multiple linear regression (RMMLR) proposed by Basu et al. (2013) and the MANOVA method. RMMLR was developed based on multivariate regression and transformed the phenotype and genotype data to achieve a rapid gene-based genome-wide association test for multiple traits. The R package that implements the method, termed as RMMLR, is available at GitHub: https://github.com/SAONLIB/RMMLR. For the MANOVA analysis, the association between each SNP in a gene and multi-trait is implemented with the MANOVA function in R. The minimum *p*-value in a gene is recorded as the gene-level *p*-value.

The genetic data were simulated to mimic the real structure of a gene through the software EpiSIM (Shang et al., 2013). The software package of EpiSIM can be downloaded at https://sourceforge.net/projects/episimsimulator/. We simulated correlated quantitative phenotypes with the following model:

$$Y_{i}=0.02\,Z_{i\,1}+0.6\,Z_{i\,2}+h\,\left(X_{i}\right)+\epsilon_{i},i=1,\ldots \,\ldots ,n,$$

where ϵ_*i*_ = (ϵ_*i*1_,ϵ_*i*2_,⋯,ϵ_*id*_)^*T*^ is a *d*-dim random error vector generated from a multivariate normal distribution with mean 0 and covariance Σ; *Y*_*i*_ = (*Y*_*i*1_,*Y*_*i*2_,⋯,*Y*_*id*_)^*T*^ is a *d*-dim-dependent trait vector; *Z*_*i*1_∼*N*(2,1) and *Z*_*i*2_∼*Ber*(0.6) are two independent covariates; *X*_*i*_ = (*X*_*i*1_,*X*_*i*2_,⋯,*X*_*ip*_)^*T*^ is a *p*-dim SNP genotype vector in a gene. Under all scenarios, we simulated genes with different dimensions, i.e., *p* = 50 and *p* = 100, and with different sample sizes, namely, *n* = 100, 200, and 400. For the number of traits, we assumed *d* = 5. The correlation between traits was assumed to be *p* = 0.3 and 0.8, with the purpose to evaluate the impact of correlation on the testing power. In each scenario, we applied 1,000 simulation replications.

We assessed the type 1 error rates under the null hypothesis [i.e., *h*(⋅) = 0] by the proportion of results that incorrectly rejected the null hypothesis. To evaluate the power, we set up four different scenarios for the *h*(⋅) function and recorded the proportion of results that rejected the null hypothesis. Under scenario A, we assumed that $h\,\left(x\right)=0.2\,\left(x_{1}-x_{6}\right)+\cos\left(x_{6}\right)\,\exp\left(-x_{6}^{2}/4\right)$, where the 1st and 6th SNP have a main effect with different directions and the 6th SNP also has a nonlinear effect on the five response traits. Under scenario B, we assumed that *h*(*x*) = 0.3*x*_2_ + 0.6*x*_4_−0.07*x*_8_.

To mimic the situation where a large number of SNPs influence the traits, we assumed the following model:

$$h\,\left(x\right)=c_{M}\,\sum_{k\in S_{M}}\alpha_{k}\,x_{k}+c_{N}\,\sum_{k,k^{\prime }\in S_{N}}\beta_{k\,k^{\prime }}\,x_{k}\,x_{k^{\prime }}$$

where *S*_*M*_ consists of a predefined set of 10 SNPs with main effect, and *S*_*N*_ contains a set of 30 SNP pairs with interactions. Both {α_*k*_,*k* ∈ *S*_*M*_} and {β_*kk*′_,(*k*,*k*′) ∈ *S*_*N*_} were generated from a uniform distribution with Unif (0, 0.02), and were fixed for all simulation replicates once generated. Under scenario C, we set *C*_*M*_ = 0.02 and *C*_*N*_ = 1.8, which gave a combination of weak main effect and relatively strong interaction effect. Under scenario D, we set *C*_*M*_ = 3.8, and *C*_*N*_ = 0, with a pure main effect model. The four scenarios with their corresponding mean functions are summarized here:

Scenario A: $h\,\left(x\right)=0.2\,\left(x_{1}-x_{6}\right)+\cos\left(x_{6}\right)\,\exp\left(-x_{6}^{2}/4\right)$
  Nonlinear effect
Scenario B: *h*(*x*) = 0.3*x*_2_ + 0.6*x*_4_−0.07*x*_8_
  Linear effect
Scenario C: $h\,\left(x\right)=0.02\,\sum_{k\in S_{M}}\alpha_{k}\,x_{k}+1.8\,\sum_{k,k^{\prime }\in S_{N}}\beta_{k\,k^{\prime }}\,x_{k}\,x_{k^{\prime }}$
  Weak main but strong interaction effects
Scenario D: *h*(*x*) = 3.8∑_*k* ∈ *SM*_α_*k*_*x*_*k*_
  Pure main effects

### Simulation Results

Table 1 displays the empirical type 1 error rate of different methods under different settings, from which we conclude that the three methods maintained reasonable type 1 error rate control in most settings.

**TABLE 1:** The type 1 error rate of different methods under different settings.

**Data dimension**	**Sample size (*n*)**	**Correlation (*p*)**	**OMGA**	**RMMLR**	**MANONA**
*p* = 50	100	0.3	0.059	0.037	0.052
		0.8	0.045	0.052	0.041
	200	0.3	0.050	0.061	0.038
		0.8	0.048	0.049	0.032
	400	0.3	0.048	0.064	0.052
		0.8	0.051	0.061	0.061
*p* = 100	100	0.3	0.044	0.052	0.046
		0.8	0.049	0.038	0.044
	200	0.3	0.061	0.041	0.046
		0.8	0.041	0.067	0.043
	400	0.3	0.051	0.057	0.035
		0.8	0.047	0.050	0.037

**FIGURE 1:**
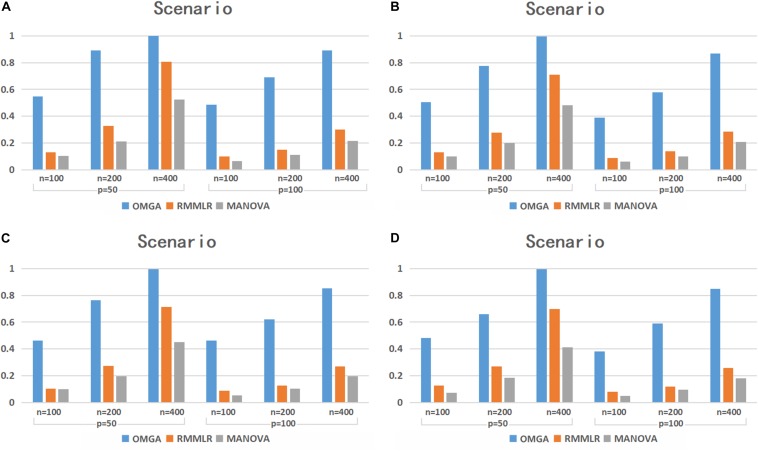
The testing power of different methods under the four scenarios with *p* = 0.3.

The power simulation results for the case with *p* = 0.3 are shown in Figure 1. Under different scenarios, the power of the three methods all increases as the sample size increases. Among the three methods, MANOVA performs the worst in most cases. Although the power decreases as the SNP dimension increases for all the three methods, the power decrease is more dramatic for RMMLR and MANOVA compared to that for OMGA. This indicates the relative advantage of the proposed method against the other two when the data dimension is high. The result clearly shows that the proposed omnibus test outperforms the other two methods under different scenarios since it can better capture the potential nonlinear effect of variants within a gene by applying a nonparametric KBT procedure with different kernel choices.

**FIGURE 2:**
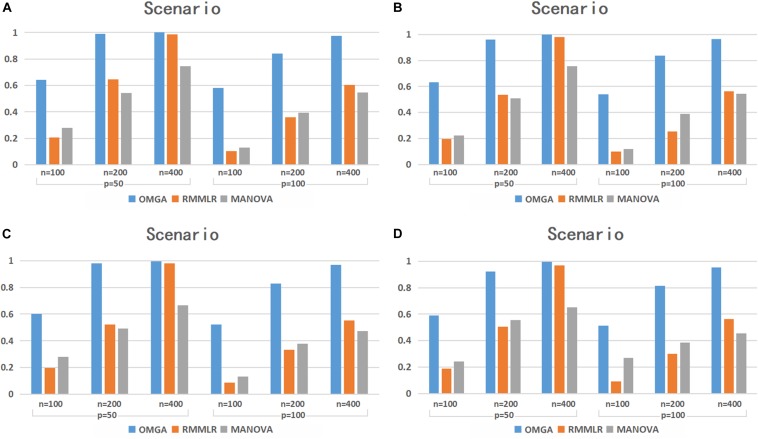
The testing power of different methods under the four scenarios with *p* = 0.8.

Figure 2 shows the empirical testing power of the three methods with *p* = 0.8. Compared with the *p* = 0.3 case, the power of RMMLR and MANONA decreased, while our proposed method can still maintain a comparable power as the *p* = 0.3 case. Note that the MANOVA method implemented here uses a minimum *p*-value approach among multiple SNPs to denote a gene-level *p*-value. The simulation result echoes the work of Basu and Pan (2011), in which the minimum *p*-value method performs the worst among the three methods that the authors compared in their simulation study.

In summary, the simulation results clearly demonstrate that the proposed omnibus test method can maintain a reasonable type I error control while having better power than the other two methods under different scenarios. This is because the proposed omnibus testing method can efficiently capture a linear or a nonlinear relationship between multiple variants in a gene and multiple phenotypes. In practice, the underlying true disease–gene relationship is never known. This makes our proposed omnibus test method particularly attractive in real application since it does not put any model assumption. As long as the choice of kernel functions is rich enough, the omnibus test can achieve its power advantage against the other methods which only function well under the desired model assumption.

## Real Data Analysis

### Case One: The Human Liver Cohort Data Analysis

To demonstrate the power and the applicability of our approach, we applied the proposed method OMGA together with RMMLR and MANONA to a HLC study data set, which can be downloaded from https://www.synapse.org/#!Synapse:syn4499. The HLC study aims to explore the genetic architecture of gene expressions in human liver. There are a total nine phenotypes of P450 enzymes (CYP1A2, 2B6, 2C8, 2A6, 2C9, 2D6, 2C19, 2E1, and 3A4) from unrelated liver samples of Caucasian individuals. The samples were removed if their genotype and phenotype information were missed, and the final data included in our study contained 170 individuals. DNAs were genotyped by the Illumina 650Y SNP and Affymetrix 500K SNP genotyping arrays. SNPs with a minor allele frequency (MAF) less than 5% were removed. The total number of SNPs that remained was 312,082, which were further mapped into 11,579 genes using tools from the NCBI website ftp://ftp.ncbi.nih.gov/snp/.

The cytochrome P450s compose a superfamily of monooxygenases which are critical for anabolic and catabolic metabolism in almost all living organisms (Nelson et al., 1996; Aguiar et al., 2005; Plant, 2007). With its importance in physiology and drug metabolism in human, the regulatory mechanisms and genetic variations of P450 enzyme have been extensively studied. As there is a relatively close relationship among the CYP family enzymes, a joint analysis of multiple P450 enzyme traits and gene association can potentially lead to the identification of novel genes. Based on a hierarchical clustering analysis, we focused on six enzyme activity traits, namely, CYP1A2, CYP3A4T, CYP2C8, CYP2B6, CYP2C9, and CYP2A6, as the response variables since they show a moderate correlation (see Supplementary Figure S1). We included age and gender as covariates in the analysis and log-transformed the six response variables.

**FIGURE 3:**
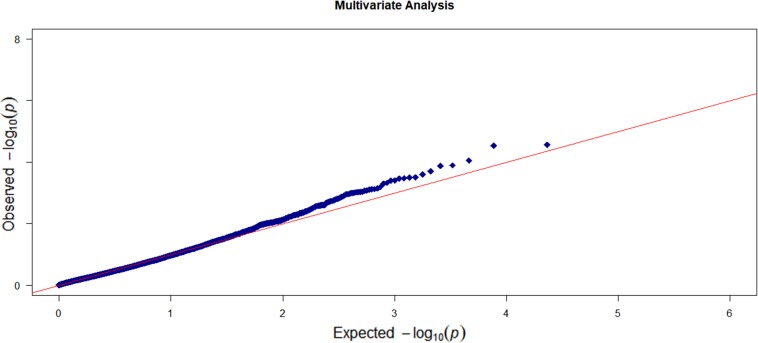
The *Q*–*Q* plot of the observed –log10 (*p*-value) versus the expected –log10 (*p*-value) for the six enzyme traits based on the multi-trait analysis.

For each individual trait, we first conducted a marginal gene-based single-trait analysis with the omnibus KBT. Then, we integrated the *p*-values for the six traits and applied the *p*-value combination method to get a gene-based multi-trait *p*-value. In the multi-trait analysis, we also applied the RMMLR and the MANONA methods. The *Q*–*Q* plot of the single-trait analysis is shown in Supplementary Figure S2 and no *p*-value inflation was observed. Figure 3 shows the *Q*–*Q* plot of the multi-trait analysis.

If we use the genome-wide gene-level Bonferroni correction, the threshold to claim a significant gene level significance is 4.3 × 10^–6^. This leads to no significant genes in our analysis. Here, we only listed a few top genes with *p*-value less than 6 × 10^–5^ as suggestive significance. In the single-trait analysis, the top genes for each trait are *HAUS8* and *IRS12* for CYP1A2, *TRAPPC10* for CYP3A4T, *TARID* and *FUNDC2* for CYP2C9, and *PAPLN* for CYP2A6. No genes pass the suggested threshold for trait CYP2B6 and CYP2C8 (see Supplementary Table S1 for a detailed list of associated genes for each trait and the corresponding *p*-values). For the multi-trait analysis, we listed in Table 2 the results of the top genes along with the results by RMMLR and MANOVA. Among the four genes, *TARID*, *TRAPPC10*, and *HAUS8* were also in the list of single-trait analysis. Gene *ATAD3C* is not shown in the top list of the single-trait analysis. This may be due to the low power of the single-trait analysis. If we ignore the correlation information among the six enzyme traits and only focus on a single-trait analysis, we may miss some discoveries. For the top four genes by OMGA, the *p*-values by RMMLR and MANOVA are all quite large. This could be due to the potential complex functional relationship between the genes and the traits. RMMLR and MANOVA were not designed to capture those complex relationships.

**TABLE 2:** List of top genes and the *p*-values with different methods in the Human Liver Cohort study.

**Gene name**	**Number of single nucleotide polymorphisms**	**Chr**	**OMGA**	**RMMLR**	**MANONA**
*TARID*	80	6	1.11E−05	0.1227	0.1048
*TRAPPC10*	58	21	1.29E−05	0.0072	0.1003
*HAUS8*	42	19	4.22E−05	0.0425	0.1022
***ATAD3C***	150	1	5.53E−05	0.0789	0.0926

Empirical evidence supports some of the identified genes. For example, gene *ATAD3C* has been reported in literature to be associated with aldosterone metabolism and P450 enzyme (Chu et al., 2017). Gene *TARID* participates in liver cell metabolism (Yuan et al., 2016). Gene *TRAPPC10* is associated with the toxic effect of octylphenol on the expression of genes in the liver (Li et al., 2014).

### Case Two: The Alzheimer Disease Neuroimaging Initiative Data Analysis

We also applied the developed OMGA method to the ADNI data set which can be accessed at http://adni.loni.usc.edu/. From the ADNI1 and ADNI2 studies, we selected 490 samples with complete genetic and phenotypic information. We deleted SNPs with MAF < 0.05 or those that could not pass the Hardy–Weinberg equilibrium test. This ended up with 620,901 SNPs. We included SNPs within 20 kb upstream and downstream of each gene and mapped them to 22,890 genes according to human genome version GRCh38.

Alzheimer’s disease (AD) is a central nervous system degenerative disease with insidious onset and chronic progress and has affected over 5.5 million Americans, especially among the elderly over the age of 65 years. ADNI provides pre-calculated volumes of five cortical regions including entorhinal, hippocampus, ventricles, midtemp, and fusiform. Brain atrophy is a typical clinical symptom among AD patients (Ferrarini et al., 2006). Studies have pointed out that the volumes in the different cortical regions show different rates of decline and are functionally related to AD. For example, the hippocampus region helps humans to deal with memory sounds, long-term learning, and taste and is a sensitive early indicator of AD (Mu and Gage, 2011). The loss in the entorhinal region is highly correlated with the severity of AD and the loss is obvious even in mild AD patients (Juottonen et al., 1998). Similarly, the volumes in the regions fusiform and midtemp also slightly decrease in AD patients (Thambisetty et al., 2011). This motivates us to take the volumes of the five cortical regions as a multi-trait response and to identify which genes are associated with the volume variation in the different brain regions.

**FIGURE 4:**
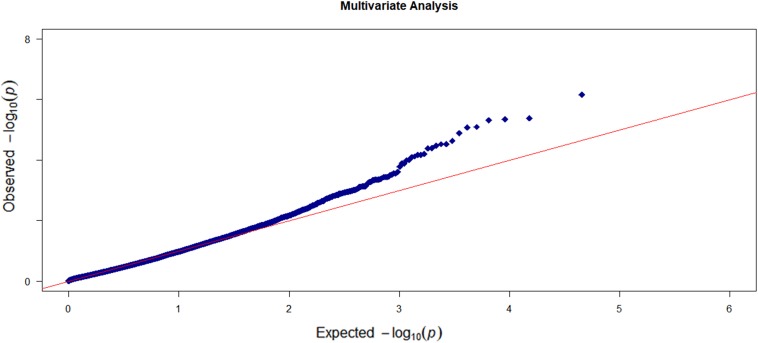
The *Q*–*Q* plots of the observed –log10 (*p*-value) versus the expected –log10 (*p*-value) for the five cortical regions based on the multi-trait analysis.

We first conducted the marginal single-trait analysis with the proposed gene-based omnibus kernel testing approach. We log-transformed the volumes of the five cortical regions and took the age, education level, gender, and *APOE4* alleles as the covariates. The *Q*–*Q* plot of the gene-based single-trait analysis is shown in Supplementary Figure S3. No sign of *p*-value inflation was observed. Also, there is no strong indication of significant signals either. Then, we carried out the multi-trait analysis which can more accurately reflect the brain atrophy in AD patients. We also applied MANOVA and RMMLR methods for multi-trait analysis. The *Q*–*Q* plot of the multi-trait analysis results by OMGA is shown in Figure 4. There is no significant indication of *p*-value inflation.

Again no significant genes were identified based on the genome-wide gene-level Bonferroni threshold. Here, we listed the top 12 genes based on a suggestive threshold of 5 × 10^–5^ in Table 3. From the single-trait analysis, we found eight, 10, 10, five, and six genes associated with the regions entorhinal, ventricles, hippocampus, fusiform, and midtemp, respectively (see Supplementary Table S2 for a detailed list of the genes). Two genes (*SNORA30* and *TLR4*) that were not in the single-trait analysis list but showed up in the multi-trait analysis list are highlighted in bold font in Table 3. Compared to RMMLR and MANOVA analyses, the *p*-values by OMGA are uniformly smaller, indicating the power of OMGA by taking both linear and nonlinear effect into consideration.

**TABLE 3:** List of top genes and the *p*-values with different methods in the Alzheimer Disease Neuroimaging Initiative study.

**Gene name**	**Number of single nucleotide polymorphisms**	**Chr**	**OMGA**	**RMMLR**	**MANONA**
*TMEM26-AS1*	731	10	3.45E−06	0.0004	0.2572
*TPRG1-AS2*	320	3	6.60E−06	0.0238	0.4595
*ST3GAL4*	2,457	11	8.37E−06	0.1373	0.0165
*LMNTD1*	89	12	9.64E−06	0.6580	0.1698
*OR4F5*	2,234	1	1.03E−05	0.1887	0.1364
*MIR6723*	170	14	1.83E−05	0.5421	0.2648
*RBM45*	468	2	2.25E−05	0.0017	0.0077
*ADAMTS7P1*	1,444	15	2.29E−05	0.0003	0.3606
*SNORA30*	200	16	2.30E−05	0.0213	0.0093
*TLR4*	153	9	3.45E−05	0.0015	0.1364
*C5orf46*	663	5	3.69E−05	0.1254	0.0232
*UPK1B*	772	3	4.10E−05	0.1855	0.0036

For the 12 genes associated with multi-trait of brain atrophy in AD patients, some of them have been reported in the literature. For example, gene *RBM45*, known as the RNA-binding motif protein 45 or developmentally regulated RNA-binding protein-1 (*Drbp1*), has been shown to be associated with the degenerative neurological changes in AD patients (Eck et al., 2018). Gene *UPK1B* has been shown to be cooperated with *CD9* and *CD81* and is directly involved in the pathological process of AD (De Strooper and Wakabayashi, 2011; Orre et al., 2014; Wężyk and Żekanowski, 2017). Mutation in gene *TLR4* reduces microglial activation, increases Aβ deposits, and exacerbates cognitive deficits in a mouse model of AD (Song et al., 2011). A study showed that polymorphisms in gene *TLR4* and *CD14* were closely related to AD (Balistreri et al., 2008). Others reported the increasing expressions of *TLR2* and *TLR4* on the peripheral blood mononuclear cells of AD patients (Zhang et al., 2012). These empirical evidences support the results of the analysis.

## Discussion

Increasing evidence has shown that, for correlated phenotypes, multi-trait analysis can significantly increase the power of association analysis (e.g., He et al., 2013; Schifano et al., 2013; Wang, 2014). Given that genes are functional units in most living organisms, we proposed a rapid and powerful gene-based multi-trait analysis method. Our method is developed under the KBT framework without specific error distribution assumptions. It possesses a few advantages over existing methods. First, the method achieves fast calculation speed and decreases the computational burden for high-dimensional data. A testing *p*-value can be quickly computed with the asymptotic results, making the method computationally attractive. Second, it can capture a potential nonlinear effect within genes by using a nonparametric KBT procedure. By incorporating different kernel functions, potential linear or nonlinear genetic effects can be captured and tested. When a given series of candidate kernel functions is available, the omnibus testing procedure is robust against misspecification of kernel functions. Moreover, it is built upon the Cauchy transformation and is computationally fast (Liu and Xie, 2019). Thus, the proposed method enjoys both theoretical rigor and computational efficiency and can be widely used in gene-based analysis.

We conducted extensive simulation studies to evaluate the type I error control and the power of the proposed method. The results show that the proposed OMGA method can maintain a reasonable type 1 error rate and achieve great power compared to other popular methods such as MANOVA and RMMLR. Furthermore, the omnibus testing procedure incorporating different kernels performs as well as if the underlying true genetic function is correctly specified. Thus, the method is safe to apply in real applications regardless of the underlying disease function, making the method practically attractive.

For multi-trait analysis, there are two different frameworks proposed. One is to jointly model multiple traits as a multivariate response and further assess their association with SNP variants. This framework can directly take correlation information into consideration. Methods for such type of multi-trait analysis include the RMMLR and the MANOVA methods as discussed in this work and many others (e.g., Maity et al., 2012]. Another framework is to conduct a single-trait disease–gene association test and then combine *p*-values to assess the joint association. The method developed by Yang et al. (2016) falls into this category. Nevertheless, methods to combining *p*-values have to take the correlation information into consideration. Otherwise, the results can be biased. Ideally, the first framework should be preferable since it models multiple traits simultaneously in one joint model. On the other hand, the second framework has its advantages. For example, it can be computationally less expensive and ease theoretical evaluations. Especially with the proposed method in this work, the second framework can be a better choice since the asymptotic evaluation of the joint association statistics can be theoretically challenging or may not even be feasible.

Our method can be easily applied to a genome-wide pathway-based multi-trait analysis. It is known that genes usually do not work alone. For example, cellular pathways and complex molecular networks are often more directly involved in the progression and the susceptibility of diseases. Thus, a pathway-based analysis can shed light on the mechanics of complex diseases. On the other hand, the current study only focused on quantitative multivariate phenotypes. It can be extended to qualitative response variables or a combination of qualitative and quantitative phenotypes. However, the extension is non-trivial and will be studied in our future investigation. The R code that implements the method can be found in GitHub at https://github.com/yamin-19/OMGA.

## Data Availability Statement

The HLC dataset can be downloaded at https://www.synapse.org/#!Synapse:syn4499. The ADNI dataset can be accessed through http://adni.loni.usc.edu/.

## Author Contributions

YD implemented the method and drafted the manuscript. TH derived the kernel testing method. RF, SL, and HC were involved in the simulation and data analysis. YC conceived the idea, designed the study, and drafted the manuscript. All the authors read and approved the final manuscript.

## Conflict of Interest

The authors declare that the research was conducted in the absence of any commercial or financial relationships that could be construed as a potential conflict of interest.
