# Supplementary material for: Genome-Wide Gene-Based Multi-Trait Analysis
Source: Front Genet. 2020 May 19;11:437. doi: 10.3389/fgene.2020.00437 (PMC7248273; doi:10.3389/fgene.2020.00437)
Supplement: Supplementary file 1 [file Data_Sheet_1.pdf]

Supplemental file for "Genome-wide gene-based multi-trait analysis"

Yamin Deng[1], Tao He[2], Ruiling Fang[1], Shaoyu Li[3], Hongyan Cao[1] and Yuehua Cui[4*]

[1]*Division of Health Statistics, School of Public Health, Shanxi Medical University, Taiyuan, Shanxi, China*

[2]*Department of Mathematics, San Francisco State University, San Francisco, CA, USA*

[3]*Department of Mathematics and Statistics, University of North Carolina at Charlotte, Charlotte, NC, USA*

[4]*Department of Statistics and Probability, Michigan State University, East Lansing, MI, USA*

*** Correspondence:** cuiy@msu.edu

Figure S1 shows the correlation between the six enzyme traits with the Pearson correlation coefficient ranging from 0.34 to 0.51.

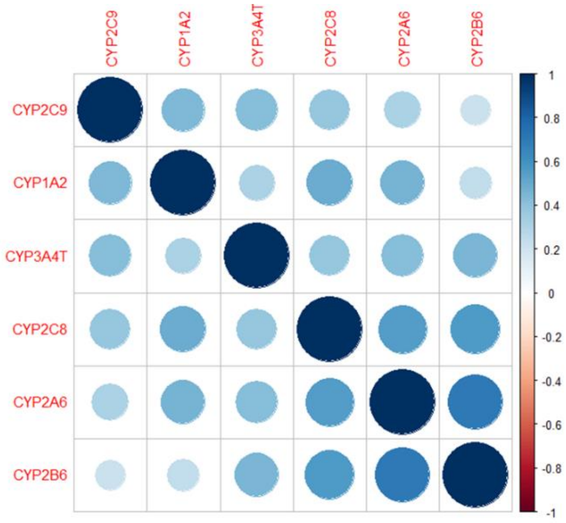

Figure S1: The Pearson correlation coefficient between the six enzyme traits (CYP1A2, CYP2C8, CYP3A4T, CYP2B6, CYP2C9 and CYP2A6).

Figure S2 shows the QQ plot of the six enzyme traits with the proposed gene-based single trait analysis. There is no indication of p-value inflation.

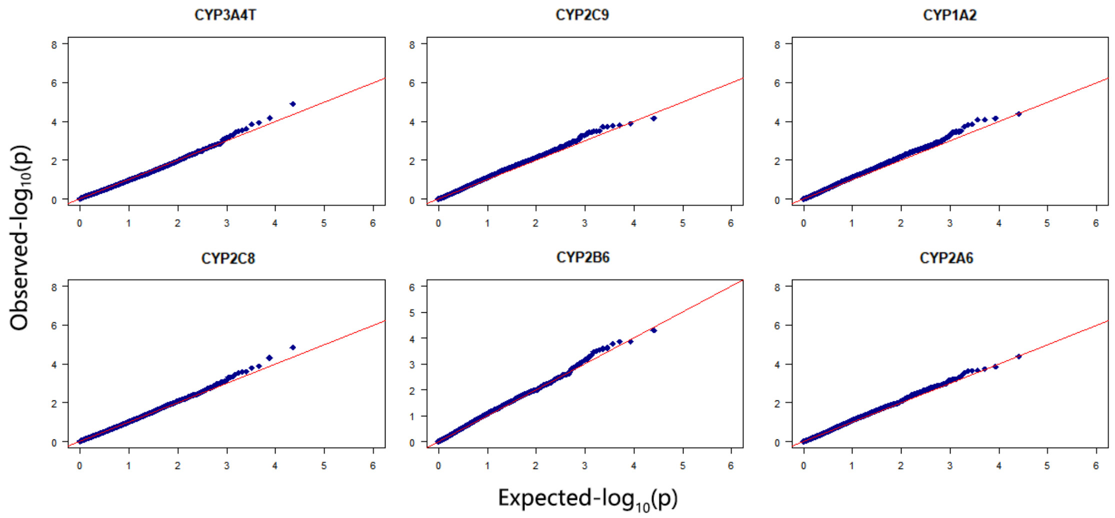

Figure S2: The Q-Q plots of the observed -log10(p-value) versus the expected -log10(p-value) for the six enzyme traits in the first case study.

Figure S3 shows the QQ plot of the five cortical regions with the proposed gene-based single trait analysis. Again, there is no sign of p-value inflation.

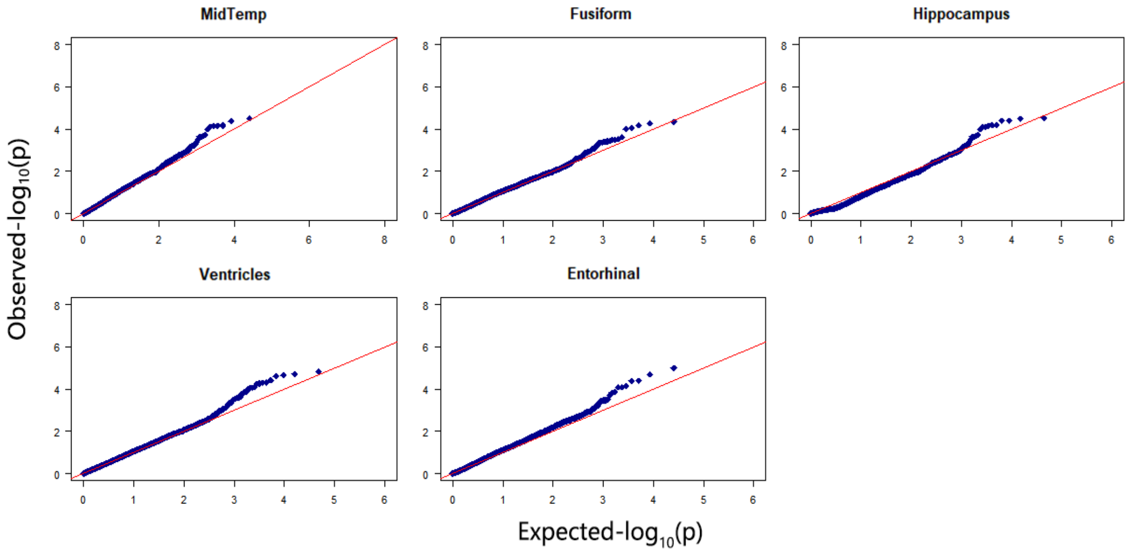

Figure S3: The QQ plots of the observed -$\log_{10}$(p-value) versus the expected -$\log_{10}$(p-value) for the five cortical regions in the 2nd case study.

Table S1 shows the genes associated with the six individual enzyme traits with the proposed gene-based single trait analysis.

Table S1. List of genes based on the single trait analysis for the six enzyme traits.

CYP1A2	CYP3A4T	CYP2B6	CYP2C9	CYP2A6	CYP2C8
HAUS8(1.44E-05)	*TRAPPC10*(5.20E-05)	0	*TARID*(1.22E-05)	*PAPLN*(3.32E-05)	0
IRS1(4.90E-05)			*FUNDC2*(6.70E-05)		

Table S2 shows the top genes associated with the five individual cortical regions traits with the proposed omnibus gene-based single trait analysis.

Table S2. List of top genes based on the single trait analysis for the five cortical regions.

Hippocampus	Entorhinal	Fusiform	MidTemp	Ventricles
TPRG1-AS2(2.99E-05)	*TMEM26-AS1*(1.00E-05)	*LMNTD1*(6.45 E-05)	*LMNTD1*(3.06E-05)	*OR4F5*(3.79E-05)
TMEM26-AS1(3.33E-05)	*HSD3B2*(2.00E-05)	*APEX1*(4.43E-05)	*APEX1*(4.32E-05)	*LOC729737*(5.61E-05)
SLBP(4.03E-05)	*HSD3B1*(4.00E-05)	*TMEM170A*(5.35E-05)	*ST3GAL4*(6.73E-05)	*LOC101928626*(1.86E-05)
ZNF689(4.04E-05)	*HS6ST3*(4.00E-05)	*ADAMTS7P1*(6.53E-05)	*ADAMTS7P1*(6.97E-05)	*MIR6723*(2.23E-05)
PRR14(6.21E-05)	*LMOD1*(7.00E-05)	*CFDP1*(8.99E-05)	*FLNC*(7.26E-05)	*LOC100133331*(2.49E-05)
BFSP1(6.63E-05)	*UGT2B10*(8.00E-05)		*OR3A1*(7.82E-05)	*LOC100288069*(5.44E-05)
LOC730183(6.82E-05)	*OR4K15*(8.00E-05)			*FAM87B*(8.17E-05)
FBRS(7.60E-05)	*FBRS*(9.86E-05)			*LINC00115*(8.56E-05)
TLR4(8.04E-05)				*LINC01128*(8.65E-05)
